# The Effectiveness and Safety of Remdesivir Use in COVID-19 Patients with Neutropenia: A Retrospective Cohort Study

**DOI:** 10.3390/life14101252

**Published:** 2024-10-01

**Authors:** Peng-Huei Liu, Ming-Wei Pan, Yan-Bo Huang, Chip-Jin Ng, Shou-Yen Chen

**Affiliations:** 1 Department of Emergency Medicine, Chang Gung Memorial Hospital, Chang Gung University College of Medicine, Taoyuan 333, Taiwan; penghuei.liu@gmail.com (P.-H.L.); yanhusuo79619@gmail.com (Y.-B.H.); ngowl@ms3.hinet.net (C.-J.N.); 2 Department of Emergency Medicine, En Chu Kong Hospital, New Taipei City 237, Taiwan; b8801136@tmu.edu.tw; 3 Graduate Institute of Management, College of Management, Chang Gung University, Taoyuan 333, Taiwan

**Keywords:** neutropenia, remdesivir, COVID-19, safety, MASCC, WHO ordinal scale

## Abstract

Background: The COVID-19 pandemic poses severe risks for immunocompromised patients, especially those with neutropenia due to chemotherapy. This study evaluates the safety and effectiveness of remdesivir use in COVID-19 patients with neutropenia. Methods: This retrospective study used the Chang Gung Research Database (CGRD) and extracted data from 98,763 patients with COVID-19 diagnosed between April 2021 and September 2022. The patients were divided into groups based on their remdesivir use and the presence of neutropenia. The adverse effects of remdesivir and their outcomes were analyzed after propensity score matching. Results: We compared common adverse effects of remdesivir in neutropenic patients before and after a 5-day regimen. A slight decrease in heart rate was observed but lacked clinical significance. There were no significant differences observed in hemoglobin, liver function tests, and blood glucose levels. After propensity score matching of COVID-19 patients with neutropenia according to gender, age, dexamethasone use, oxygen use, MASCC score, and WHO ordinal scale, no significant differences were found in length of stay, intubation rate, or ICU admission rate between the matched patients. Conclusions: Our study found remdesivir to be safe for COVID-19 patients with neutropenia, with no common adverse reactions observed. However, its effectiveness for these patients remains uncertain.

## 1. Introduction

The coronavirus disease 2019 (COVID-19) rapidly spread globally starting in 2019, causing a pandemic. The clinical manifestations range from asymptomatic to respiratory failure and even death [1]. Particularly, patients with diabetes, chronic lung diseases, heart diseases, chronic kidney disease, transplant recipients, and malignancy are more prone to severe illness [2]. For patients with malignant tumors, the use of chemotherapy drugs in cancer treatment can lead to febrile neutropenia, which not only increases the risk of COVID-19 complications but also makes bacterial or fungal infections more likely. Therefore, finding effective and safe treatments for COVID-19 in these immunocompromised patients is extremely important [3].

Treatments for COVID-19 are constantly evolving, and many organizations have provided treatment guidelines for severe cases requiring oxygen, including dexamethasone, remdesivir, baricitinib, and tocilizumab [4,5,6]. Among them, remdesivir is a viral RNA-dependent polymerase inhibitor, and some studies have shown that it can shorten the recovery time of hospitalized adult patients with COVID-19 who have evidence of lower respiratory tract infection and also reduce the risk of requiring oxygen during the early stages of hospitalization [7,8,9]. For critically ill patients in the ICU, early use of remdesivir can also provide a survival benefit [10,11,12]. However, remdesivir is known to cause some side effects, such as nausea, vomiting, elevated transaminase levels, anemia, and hyperglycemia, and in certain cases, it may lead to bradycardia [7,13,14]. Cancer patients undergoing chemotherapy especially are more prone to anemia due to the side effects of bone marrow suppression compared to the general population. Additionally, certain chemotherapy drugs (such as methotrexate, asparaginase, or carmustine) can also lead to abnormal liver function. Therefore, there are some concerning situations when using remdesivir [15]. Therefore, our study aims to evaluate the safety and effectiveness of remdesivir in the population with COVID-19 infection combined with neutropenia.

## 2. Material and Methods

### 2.1. Study Design and Data Source

The present study is a retrospective cohort investigation that utilized electronic health records (EHR) sourced from the Chang Gung Research Database (CGRD) [16]. The database is de-identified and originates from the EHRs of Chang-Gung Memorial Hospital (CGMH), which is one of the largest healthcare systems in Taiwan. CGMH consists of seven medical institutions spread across Taiwan, serving the country’s population of 23 million. It provides more than 10,000 beds and offers medical care to more than 280,000 patients per year. Since 2000, the CGRD has been compiling EHRs of all patients without the necessity for a specific registration process at CGMH. The database contains a wide range of information, including demographics, laboratory data, inpatient data, outpatient data, emergency patient data, nursing data, disease category data, and treatment data including hospital medication and procedures.

This study obtained approval from our institutional review board (IRB no. 202401139B0).

### 2.2. Study Setting and Population

Between April 2021 and September 2022, Taiwan experienced various stages of COVID-19 variant outbreaks. In April 2021, the Alpha variant (B.1.1.7) began to spread, leading to a domestic outbreak in May, prompting the government to implement Level 3 pandemic control measures. As the pandemic progressed, the Delta variant (B.1.617.2) became the dominant strain from mid-2021 to the end of the year, with higher transmissibility leading to increased hospitalization and severe cases. However, starting in early 2022, the Omicron variant (BA.1 and BA.2 subvariants) rapidly replaced Delta as the primary circulating strain.

We retrieved data from the CGRD on COVID-19 patients who visited the emergency department (ED) from April 2021 to September 2022. In this group of COVID-19 patients, we included patients with neutropenia (absolute neutrophil count (ANC) < 500/uL) and excluded patients under 18 years of age or those who did not need admission. We retrieved basic demographics, including age, gender, laboratory data, vital signs, and underlying diseases, and the data of treatment and prognostic factors such as mortality, length of stay (LOS), intensive care unit (ICU) admission, and use or not of intubation. Additionally, we calculated the World Health Organization (WHO) ordinal scale and Multinational Association of Supportive Care in Cancer (MASCC) score of patients to analyze the severity of COVID-19 infection in patients with neutropenia [17,18].

The WHO Ordinal Clinical Severity Scale classifies COVID-19 patients’ clinical status from 0 to 8. The scale’s nine levels are as follows: 0—Uninfected: No clinical or virological evidence of infection; 1—Ambulatory: No limitation of activities; 2—Ambulatory: Limitation of activities; 3—Hospitalized: No oxygen therapy; 4—Hospitalized: Oxygen by mask or nasal prongs; 5—Hospitalized: Non-invasive ventilation or high-flow oxygen; 6—Hospitalized: Intubation and mechanical ventilation; 7—Hospitalized: Ventilation and additional organ support (e.g., pressors or extracardiac membranous oxygenation); 8—Death [17].

The MASCC risk index score is applied to identify febrile neutropenia patients with low risk who can be managed with outpatient care. The MASCC risk index score assesses various clinical factors and assigns points based on the patient’s condition. The total score determines the patient’s risk level. The MASCC risk index score includes the following criteria: Burden of Illness (no or mild symptoms): 5 points; No Hypotension (systolic blood pressure > 90 mm Hg): 5 points; No Chronic Obstructive Pulmonary Disease (COPD): 4 points; Solid Tumor or Hematologic Malignancy with No Previous Fungal Infection: 4 points; No Dehydration Requiring Parenteral Fluids: 3 points; Burden of Illness (moderate symptoms): 3 points; Outpatient Status at the Onset of Fever: 3 points; Age < 60 years: 2 points. Patients with a total score of 21 or higher are considered low-risk and can often be managed safely as outpatients. Those with lower scores may require inpatient care due to a higher risk of complications [18,19].

### 2.3. Outcome Measures

To evaluate the safety of remdesivir use in neutropenic patients, we analyzed the adverse effects in COVID-19 patients with neutropenia who received a 5-day treatment course of remdesivir. Previous studies have reported some adverse reactions to remdesivir, including bradycardia, anemia, blood glucose instability, and liver function deterioration [7,13,14]. These adverse reactions could affect patient conditions and prognosis, so these side effects were evaluated for safety in our study.

To assess the effectiveness of remdesivir treatment in COVID-19 patients with neutropenia, we compared treatment outcomes between the patient group receiving remdesivir and the patient group without remdesivir use. This comparative analysis encompassed factors such as hospital LOS, ICU admission rates, intubation status, and mortality rates. According to the treatment guidelines for remdesivir use in COVID-19 patients, the utilization of oxygen and steroids may vary between patients who received remdesivir and those who did not. To evaluate the effectiveness of remdesivir in COVID-19 patients with neutropenia, the analysis was carried out after patient matching for covariates such as age, gender, granulocyte colony-stimulating factor (G-CSF) usage, oxygen, dexamethasone usage, MASCC score, and WHO ordinal scale.

### 2.4. Statistical Analysis

We conducted data analysis using IBM SPSS Statistics for Windows (Version 24.0; IBM Corp., Armonk, NY, USA). We have checked the normality of the variables by using Shapiro–Wilk test for small sample sizes and Kolmogorov–Smirnov test for larger samples. The factors were compared by using Student’s *t*-test or Wilcoxon signed-rank test, depending on the results of normality, for continuous variables, and Pearson’s chi-square test for categorical variables. We performed one-to-one matching using propensity scores to compare the effectiveness of remdesivir in patients with neutropenia to minimize bias caused by individual differences. Statistical significance was set at *p* < 0.05.

## 3. Results

**Figure 1:**
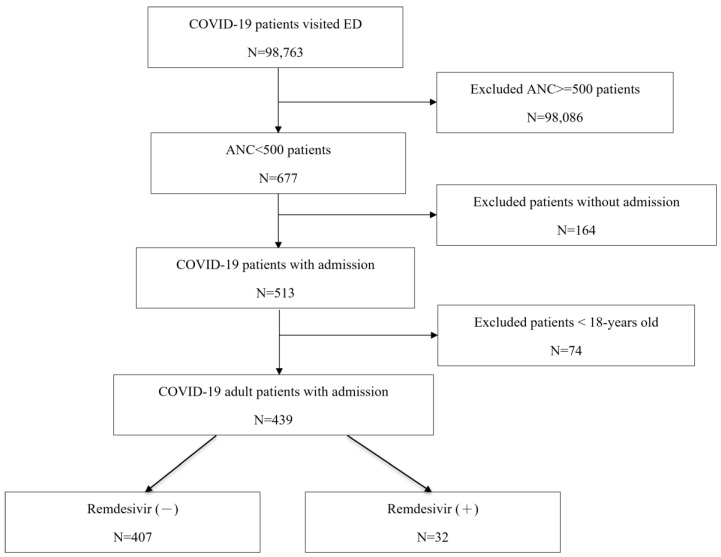
Flow diagram of patient selection.

Between April 2021 and September 2022, a total of 98,763 confirmed COVID-19 patients were included from the database, of which only 677 patients had neutropenia when they visited the ED (Figure 1). Excluding patients under the age of 18 and patients who did not require hospitalization, a total of 439 adult COVID-19 patients with neutropenia were included. Patients were further divided into those who received remdesivir treatment (N = 32) and those without remdesivir use (N = 407).

Table 1 presents the data and comparison between patient groups of usage and non-usage of remdesivir. The patient group without remdesivir was younger (63.69 years vs. 70.88 years, *p* = 0.009). Patients who received remdesivir treatment had higher rate of dexamethasone usage (12.53% vs. 78.13%, *p* < 0.001). There were no significant differences in triage vital signs and initial laboratory tests between these two patient groups at ED.

For the analysis concerning the safety of remdesivir use, we compared various parameters according to common adverse effects of remdesivir before and after a 5-day regimen in the neutropenic patients who received remdesivir treatment (Table 2). After the use of a 5-day regimen of remdesivir, a slight decrease in heart rate was observed (108 vs. 90 beats per minute, *p* < 0.001). No significant differences were found in hemoglobin (10.6 vs. 10.7 g/dL, *p* = 0.792), ALT (27 vs. 27 U/L, *p* = 0.215), and blood glucose levels (124 vs. 121 mg/dL, *p* = 0.811) after 5-day remdesivir use.

Table 3 shows the analysis of the effectiveness of remdesivir in these neutropenic patients after propensity score matching according to gender, age, dexamethasone use, oxygen use, MASCC score, and WHO ordinal scale. In order to achieve one-to-one matching of patients, the number of people in the non-remdesivir group was significantly reduced to match that of the remdesivir group. In the matched patients, no significant differences were found in LOS (12.5 vs. 14.5 days, *p* = 0.896), intubation rate (28.57 vs. 14.29%, *p* = 0.329), ICU admission rate (17.86 vs. 0%, *p* = 1), and mortality rate (35.71 vs. 21.43%, *p* = 0.375) between the two patient groups.

## 4. Discussion

Compared to patients without malignancy, patients with malignancy infected with COVID-19 have a higher risk of being admitted to the ICU, requiring intubation, and experiencing higher mortality rates [20,21]. Immunosuppression in cancer patients stems not only from the malignancy itself but also from the effects of chemotherapy. Chemotherapy-induced immune suppression can lead to febrile neutropenia, which predisposes patients to bacterial or fungal infections and increases the risk of COVID-19 complications [3]. In addition, some case reports have shown that COVID-19 may cause neutropenia in non-cancer patients, although the exact cause remains unclear. This condition could be an extremely rare complication of COVID-19 infection. [22,23]. For COVID-19 patients with neutropenia, secondary bacterial infection or complication could occur, so identifying effective treatment strategies for this population may be crucial. However, there is limited research on COVID-19 infection combined with neutropenia. Our study may be the first study to evaluate the effectiveness of remdesivir treatment in this population.

Although previous randomized controlled studies have not consistently demonstrated significant effectiveness of remdesivir for hospitalized COVID-19 patients, it has been found to shorten hospital LOS, reduce the risk of ventilation, and lower mortality rates in severe patients not receiving ventilation therapy [7,8,13,24]. Guidelines from Infectious Diseases Society of America (IDSA), National Institutes of Health (NIH), and WHO also recommend treatment with remdesivir for the treatment of severe COVID-19 [4,5,6]. Remdesivir also holds potential benefits for certain populations. In a large database study from the United States, lower 28-day mortality was observed in immunocompromised patients hospitalized for COVID-19 who received remdesivir within the first two days of admission compared to those who did not receive remdesivir treatment [25]. Given the unclear benefit for subgroups, our study further analyzed the effectiveness and side effects of remdesivir for neutropenic patients.

In previous research, common side effects associated with remdesivir included bradycardia, anemia, unstable glucose level, and deteriorated liver function [7,14]. In our study, no significant adverse reactions were observed after 5-day remdesivir treatment. A mild decrease in heart rate was observed after 5-day remdesivir use. This could be caused by the adverse effect of remdesivir or the improved clinical status. However, no clinical intervention was necessary for this heart variation. Due to the bone marrow suppression caused by chemotherapy, patients undergoing chemotherapy typically have lower hemoglobin levels compared to the general population. This may explain why the use of remdesivir did not result in a significant decrease in hemoglobin levels. Other common side effects of remdesivir were not found in these neutropenic patients who received remdesivir use. According to our results, the use of remdesivir in COVID-19 patients with neutropenia appears to be safe.

Our study failed to demonstrate the effectiveness of remdesivir in COVID-19 patients with neutropenia. One possible explanation is that COVID-19 is a viral infection, and neutrophils are not the primary immune mechanism against viral infections. In these patients, the most common cause of infection is translocation of gut bacteria [26]. Pneumonia or the need for oxygen might be due to secondary bacterial infections caused by neutropenia [27], which is why the clinical effectiveness of remdesivir is not apparent. On the other hand, some studies have indicated that neutropenia during COVID-19 is not an independent risk factor for poor outcomes in COVID-19 [28]. An excessive immune response drives the progression of COVID-19, and some patients experience rapid clinical deterioration, leading to extensive infiltration of monocytes, macrophages, and neutrophils into the lungs [29,30]. Patients with neutropenia may have less risk of excessive immune response during COVID-19 infection.

Another possible explanation was the limited number of patients included in our study. According to treatment guidelines in Taiwan, remdesivir could only be used in COVID-19 patients requiring oxygen therapy or with pneumonia proven from chest films, which resulted in a lesser number of included patients [31]. Further study including more patients may be necessary to explore the effectiveness of remdesivir use in COVID-19 patients with neutropenia.

### Limitations

This study has several limitations. First, it is a single-country, multicenter study, so selection bias could exist, and the generalizability of the results could be limited. Second, our database does not contain information on patients’ use of granulocyte colony-stimulating factor (G-CSF), so we cannot determine whether the use of G-CSF affects the prognosis of neutropenia patients with COVID-19. Third, our database lacks certain data related to severity or outcome assessment, such as presenting symptoms, lung imaging, and polymerase chain reaction (PCR) cycle threshold (CT) values. This may affect the thoroughness of our outcome assessment. Lastly, the number of included cases after matching was insufficient, leading to a lack of significant differences in the prognosis analysis.

## 5. Conclusions

This study demonstrated that it is safe to use remdesivir in COVID-19 patients with neutropenia. Adverse reactions of remdesivir in previous reports, including hyperglycemia, bradycardia, and liver function abnormalities, were not noted. A decrease in hemoglobin level was found, but clinical significance was minimal. While the use of remdesivir in COVID-19 patients appeared to have some clinical benefits in previous studies, the effectiveness remained unclear for COVID-19 patients with neutropenia in our study.

## Figures and Tables

**Table 1:** Comparison between usage and non-usage of remdesivir in COVID-19 patients with neutropenia.

	Non Remdesivir (N = 407)	Remdesivir (N = 32)	*p*-Value
Age, years, mean (SD)	63.69 (14.95)	70.88 (14.71)	0.0091 *
Gender, male, N (%)	231 (56.76)	18 (56.25)	1
Dexamethasone use, N (%)	51 (12.53)	25 (78.13)	<0.0001 *
Oxygen use, N (%)	156 (38.33)	17 (53.13)	0.1439
G-CSF use, N (%)	103 (25.31)	6 (18.75)	0.5391
Triage vital signs			
BT, °C, median (IQR) ^#^	37 (36.2–38)	37.8 (37–38.3)	0.0062 *
SBP, mmHg, median (IQR) ^#^	121 (102–139)	121.5 (106–136)	0.9032
DBP, mmHg, median (IQR) ^#^	70 (58–81)	72 (59–82)	0.8801
RR, breaths per minute, median (IQR) ^#^	18 (16–20)	20 (18–22.5)	0.0284 *
HR, bpm, median (IQR) ^#^	110 (94–124)	108 (97.5–122)	0.8958
SpO2, %, median (IQR) ^#^	96 (93–97)	91.5 (86–95)	0.0005 *
Initial laboratory data			
WBC, 1000/uL, median (IQR) ^#^	3.6 (2.1–5.3)	4.25 (2.1–5.85)	0.5784
ANC, median (IQR) ^#^	235.2 (114.8–357.5)	344.8 (160.9–437.9)	0.1013
Hb, g/dL, median (IQR) ^#^	10.2 (8.7–12.2)	10.6 (9.3–11.85)	0.4932
Creatinine, mg/dL, median (IQR) ^#^	1 (0.67–1.75)	1.1 (0.65–1.78)	0.7113
ALT, U/L, median (IQR)	26.5 (17–47)	27 (15–32)	0.2700
Bilirubin, mg/dL, median (IQR) ^#^	0.7 (0.5–1.1)	0.65 (0.4–0.8)	0.1184
CRP, mg/L, median (IQR) ^#^	90.14 (39.24–187.87)	153.29 (64.02–238.13)	0.1734
Sugar, mg/dL, median (IQR) ^#^	127 (106–160)	124 (107–164)	0.8388
Na, mEq/L, median (IQR) ^#^	134 (130–137)	133 (124–136)	0.1381
K, mEq/L, median (IQR) ^#^	3.8 (3.4–4.2)	4.1 (3.6–4.4)	0.0916
Troponin I, ng/mL, median (IQR) ^#^	0.02 (0.01–0.06)	0.01 (0.01–0.02)	0.8482
D-dimer, ng/mL, median (IQR) ^#^	3178 (1607–10,000)	-	-
Prognosis			
LOS, days, median (IQR) ^#^	12 (7–25)	14.5 (7–21)	0.9809
Intubation, N (%)	76 (18.67)	5 (15.63)	0.8482
ICU admission, N (%)	40	<3	0.9616
Mortality, N (%)	69 (16.95)	7 (21.88)	0.6413
Underlying diseases			
Cardiovascular disease, N (%) (PVD)	26 (6.39)	0 (0)	1
Hypertension, N (%)	187 (45.95)	19 (59.38)	0.1999
Congestive heart failure, N (%) (CHF)	36 (8.85)	4 (12.5)	0.3311
Cerebrovascular disease, N (%) (CD)	73 (17.94)	8 (25)	0.4501
Chronic pulmonary disease, N (%) (CPD)	47 (11.55)	4 (12.5)	0.5238
Diabetes mellitus, N (%)	122 (29.98)	14 (43.75)	0.1544
Malignancy, N (%)			0.0010
Hematology malignancy	57 (14)	4 (12.5)	
Other malignancy	234 (57.49)	9 (28.13)	
Non-Malignancy	116 (28.5)	19 (59.38)	
Renal Disease, N (%)	85 (20.88)	10 (31.25)	0.2509
Scoring systems			
MASCC score, median (IQR) ^#^	21 (16–23)	18.5 (16–21)	0.0347 *
WHO ordinal scale, median (IQR) ^#^	3 (3–4)	4 (4–5)	0.0006 *
Inflammation risk categories, N (%)			0.8562
H	240 (58.97)	19 (59.38)	
I	103 (25.31)	7 (21.88)	
L	64 (15.72)	6 (18.75)	

^#^ Wilcoxon signed-rank test, * *p* < 0.05. G-CSF: granulocyte colony-stimulating factor; BT: body temperature; SBP: systolic blood pressure; DBP: diastolic blood pressure; RR: respiratory rate; HR: heart rate; bpm: beats per minute; WBC white blood cell; ANC: absolute neutrophil count; Hb: hemoglobin; ALT: alanine transaminase; LOS: length of stay; ICU: intensive care unit; MASCC: Multinational Association of Supportive Care in Cancer; IQR: interquartile range; WHO: World Health Organization.

**Table 2:** Comparison of adverse effects of remdesivir after the usage.

	Before Remdesivir	After Remdesivir	*p*-Value
HR, bpm, median (IQR)	108 (97.5–122)	90 (79–102)	0.0003
Hb, g/dL, median (IQR)	10.6 (9.3–11.85)	10.7 (9.5–11.8)	0.7917
Bilirubin, mg/dL, median (IQR)	0.65 (0.4–0.8)	0.45 (0.3–0.6)	0.0058
ALT, U/L, median (IQR)	27 (15–32)	27 (13–45)	0.2154
Sugar, mg/dL, median (IQR)	124 (107–164)	121 (98–218)	0.8114

HR: heart rate; bpm: beats per minute; IQR: interquartile range; Hb: hemoglobin; ALT: alanine transaminase.

**Table 3:** Comparison between usage and non-usage of remdesivir in COVID-19 patients with neutropenia after propensity score matching.

	Non Remdesivir (N = 28)	Remdesivir (N = 28)	*p*-Value
Characteristics			
Age, years, mean (SD)	69.25 (14.38)	68.79 (13.94)	0.9028
Gender, male, N (%)	14 (50)	16 (57.14)	0.7887
Dexamethasone use, N (%)	21 (75)	21 (75)	1
Oxygen use, N (%)	18 (64.29)	15 (53.57)	0.5870
G-CSF use, N (%)	10 (35.71)	6 (21.43)	0.3749
Scoring systems			
MASCC score, median (IQR) ^#^	19 (16.5–21)	19 (16–21)	0.5467
WHO ordinal scale, median (IQR) ^#^	4 (3–4)	4 (4–5)	0.4219
Malignancy, N (%)			0.0945
Hematology malignancy	<3	4	
Other malignancy	16	8	
Non-Malignancy	10	16	
Outcomes			
LOS, days, median (IQR) ^#^	12.5 (5.5–30)	14.5 (7–21)	0.8956
Intubation, N (%)	8 (28.57)	4 (14.29)	0.3286
ICU admission, N (%)	5 (17.86)	0 (0)	1
Mortality, N (%)	10 (35.71)	6 (21.43)	0.3749

G-CSF: granulocyte colony-stimulating factor; MASCC: Multinational Association of Supportive Care in Cancer; IQR: interquartile range; WHO: World Health Organization; LOS: length of stay; ICU: intensive care unit. ^#^ Wilcoxon signed-rank test.

## Data Availability

The data used in this study were obtained from the Chang Gung Research Database. All analyses were conducted in an isolated space. Data regarding the analytical process and original results are available from the corresponding author upon reasonable request.
